# Low frequency of human papillomavirus infection in conjunctival squamous cell carcinoma of Mexican patients

**DOI:** 10.1186/1750-9378-6-24

**Published:** 2011-11-18

**Authors:** Raúl Peralta, Alejandra Valdivia, Perla Estañol, Vanessa Villegas, Carolina Pimienta, Eugenio Treviño, Daniel Marrero, Monica Mendoza, Florinda Jimenez, Leonardo Villalvazo, Miriam Tejeda, Mauricio Salcedo

**Affiliations:** 1 Laboratorio de Oncogenomica, UIMEO, Hospital de Oncología, CMN-SXXI, IMSS. Av Cuauhtemoc 330, Col. Doctores, 06720, Mexico DF, Mexico; 2 Servicio de Oftalmología, Fundación Hospital Nuestra Señora de la Luz I.A.P. Ezequiel Montes #135 C.P. Del. Cuauhtémoc, 06030, México, DF, Mexico; 3 Laboratorio de Genómica Funcional, Universidad Autonóma de Ciudad Juárez, Av. Plutarco Elias Calles No.1210, Fovissste Chamizal, Cd. Juarez, Chihuahua, 32310, Mexico; 4 Departamento de Patología, Instituto de Oftalmología, Fundación Conde de Valenciana, Chimalpopoca No.14 Col. Obrera, 06800, Mexico DF, Mexico; 5 Departamento de Orbita, Instituto de Oftalmología, Fundación Conde de Valenciana, Chimalpopoca No.14 Col. Obrera, 06800, Mexico DF, Mexico

**Keywords:** conjunctiva, carcinoma, HPV

## Abstract

**Background:**

The relationship between Human Papillomavirus (HPV) infection and conjunctiva cancer is controversial. HPV detection will provide more information about the role of this infectious agent in the biology of conjunctiva cancer. In the present study, DNA extracted and purified from 36 Conjunctival Squamous Cell Carcinomas (CSCC) was evaluated by PCR for HPV DNA sequences. The results were correlated with the clinical and histopathological variables.

**Results:**

The results showed that HPV DNA was present in 8 CSCC samples (22%); HPV16 was the sole type detected. Significant association was found between HPV detection and the limbus tumor subtype (p = 0.03). All the samples were non-metastatic squamous cell carcinoma.

**Conclusions:**

The HPV presence in CSCC from Mexican patients is not a common event.

## Background

Squamous cell carcinoma (SCC) is the most common neoplasm of the conjunctiva [1]. The etiology of cancer of the conjunctiva appears to be multifactorial; several risk factors have been identified, such as smoke, Human immunodeficiency virus (HIV) infection, Ultraviolet (UV) light, history of pterygium, or Human papillomavirus infection (HPV) [2,3]. However, the reports on HPV and conjunctival neoplasms are controversial. Some studies have reported a heterogeneous prevalence of high-risk HPV types, suggesting that only a subset of cases can be attributed to these viruses [4,5]. Differences in detection methods, populations, or geographic distribution could contribute to the variation in HPV infection rates in Conjunctival Squamous Cell Carcinoma (SCC)[6-12]. HPV is considered the main etiologic agent in proliferative ocular surface, lachrymal sac lesions, and pterygium worldwide, and it is has been suggested that HPV types 16 and 18 play a critical role in the oncogenesis of conjunctival cancers [13-17].

HPV are a group of host-specific DNA viruses with oncogenic subtypes that have been shown to act as carcinogens in the development of Cervical Cancer (CC), anogenital, head and neck, and CSCC [18,19]. While the E6 oncoprotein, encoded by HPV16 or HPV18, is known to bind the cancer gene product *p53 *and to promote its degradation, the E7 oncoprotein binds to the retinoblastoma cancer gene product *pRB *and results in E7-induced *pRB *inactivation. The E5 virus protein cooperates with E7 to transform cells and enhances the ability of E7 to induce proliferation, and with E6, to immortalize cells [20]. Preclinical and clinical studies suggest that the adoption of HPV vaccination strategies may exert an impact on the incidence of CC and potentially on other HPV infection-associated cancers, such as conjunctival cancer [21].

In order to elucidate the presence of HPV in CSCC, in the present study a group of fixed and paraffin-embedded specimens from Mexican patients with CSCC were analyzed, and a systematic review of the literature was performed.

## Results

### Prevalence of HPV Infection in Conjunctival Carcinoma Worldwide

The PubMed database (National Library of Medicine, Bethesda, MD, USA) was used to identify all of the articles published between 1986 and August 2011 containing combinations of the Medical Subject Headings "Human papillomavirus" and "Conjunctival carcinoma". All articles reporting data of HPV prevalence in conjunctival carcinomas were selected and reviewed (45 reports). To have a more robust analysis, publications containing < 10 studied cases were excluded from the analysis. Finally, 21 articles were included in this review. Information on country of origin, year, authors, sample size, and mucosal and cutaneous HPV prevalence was retrieved (Table 1).

**Table 1:** Geographic distribution of Human Papillomavirus in conjunctiva carcinoma

Continent	Authors	Year	N cases	HPV cutaneous prevalence	HPV mucosal prevalence	Countries
African	Yu JJ	2010	11	0	9 (81%)	Uganda, Kenya
	Ateenyi-Agaba C	2010	94	42 (44.7%)	6 (6.4%)	Uganda
	DeKoning MN	2008	24	10 (42%)	3 (13%)	Uganda
	Tornesello ML	2006	29	3 (10.3%)	0	Uganda
	Moubayeb P	2004	14	0	5 (35%)	Tanzania
	Ateenyi-Agaba C	2004	21	0	18 (86%)	Uganda
	Newton R	2002	39	0	11 (28%)	Uganda
	Waddell KM	1996	20	0	7 (35%)	Uganda, Malawi
Europe	Reszec J	2010	38	0	2 (5.2%)	Poland
	Guthoff R	2009	31	0	0	Germany
	Reszec J	2005	11	0	1 (9%)	Poland
	Toth J	2000	23	0	5 (22%)	Hungary
Asia	Manderwad GP	2009	48	0	0	India
	Sen S	2007	30	0	0	India
	Jung SM	2006	13	0	0	Taiwan
	Tulvatana W	2002	30	0	0	Thailand
	Eng HL	2002	20	0	0	China
	Karcioglu ZA	1997	31	0	17 (55%)	Saudi Arabia
Oceania	Tabrizi SN	1997	88	0	34 (39%)	Australia
America	Palazzi MA	2000	31	0	4 (12%)	Brazil
	McDonell JM	1992	11	0	7 (63%)	USA
	Present work	2011	36	0	8 (22%)	Mexico

Publications containing less than ten cases studied were excluded from the analysis. In total were 21 reports covering more than 650 cases with an average of 28% in 15 countries. A range from 0-86% was observed

### Detection of HPV Sequences in Conjunctival Carcinomas of Mexican Patients

**Figure 1:**
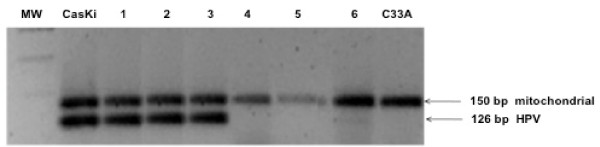
**Illustrative example of PCR reaction for the HPV16/E6 region and D-loop mitochondrial region in conjunctival carcinoma samples**. Lanes 1, 2, 3 and 6 correspond to positive HPV16 samples (126 bp); lanes 4 and 5 correspond to negative HPV16 samples; all the samples were positive to mitochondrial region amplification (150 bp). Positive HPV16 (Caski) and negative HPV (C33) controls were included. MW: molecular weight marker 100 bp.

To demonstrate the quality of the DNA for amplification reaction, DNA purified from formalin-fixed tissue samples was first subjected to PCR utilizing D-loop mitochondrial primers. This amplicon was consistent in all cases (Figure 1). Therefore, DNA was analyzed for the presence of HPV16/E6 (primers yielding a 126 bp fragment). These primer sets allowed detection of HPV DNA in 22% of the CSCC samples (8/36). Even when excessive DNA fragmentation was due to the tissue fixation process, our results might be considered as valid in showing the prevalence of HPV in the CSCC analyzed. In general, HPV16 was the sole type detected in CSCC samples (8/36, 22%). HPV type was confirmed by DNA sequencing (data not shown).

### Correlation Between HPV Detection and Clinicopathological Variables

The age of patients ranged from 24-92 years, with a mean age of 66 years. Distribution by age group was as follows: 25% (*n *= 9), > 80 years of age; 61% (*n *= 22), aged between 50 and 79 years, and only 14% (*n *= 5) were < 49 years of age. Patients positive or negative to HPV infection were stratified by age group (Table 2).

**Table 2:** Correlation between HPV detection and clinicopathological variables in conjunctival cancer samples.

Clinicopathological variables		HPV		
	N	Positive	Negative	P value
All cases	36	8	28	
**Age**				0.36
≤ 50	9	3	6	
> 50	27	5	22	
**Gender**				0.84
Male	26	6	20	
Female	10	2	8	
**Subtype Clinical**				0.03*
Limbus	21	2	19	
Conjunctive	15	6	9	
**Histological differentiation**				0.49
Well	10	3	7	
Moderate/Poor	26	5	21	

HPV: human papillomavirus presence. * Statistical significance

Anatomical analysis showed that in CSCC, limbus type was 58% (21/36), followed by conjunctival type, 42% (Table 2). None of the patients had a previous history of pterygium. Sexual transmitted diseases (STD) history was not considered because the STD data provided was not considered accurate. Significant association was found between HPV detection and limbus subtype (*p *< 0.05), but no statistically significant correlation between HPV and differentiation degree was found (*p *> 0.05) (Table 2).

## Discussion

Development of CSCC such as cervical cancer could be strongly linked with infection by high-risk HPV types [22]. There is molecular evidence that viral oncoproteins E6 and E7, found in high-risk HPV genotypes that are inactive cancer genes *p53 *and *pRb*, promote genomic rearrangement and confer replicative and immortalizing activities on cervical neoplasms and other cancers [20]. According to the model of cervical carcinogenesis, which assigns HPV infection a central initiating role, identical or similar mechanisms could be responsible for the development of conjunctival cancer.

In reviewing the literature on CSCC and its relationship with HPV, it is evident that a major discrepancy exists among the reports published. Intriguingly, in published data on HPV DNA analysis in conjunctival carcinoma, < < 1,000 samples were studied in nearly 21 reports, showing serious differences. Based on the present study and certain other reports that failed to demonstrate the HPV DNA of any HPV (mucosal or not), as in studies in Taiwan, Thailand, China, India, or Germany, we tend to conclude that HPV is highly, but not conclusively, unlikely to play any causal role in the pathogenesis of conjunctival cancer [6,8,13,15,23-25]. Even when HIV infection could act as an important risk factor for conjunctival cancer, reports showing a high percentage of HPV DNA sequences in CSCC HIV+ could indicate a biased result with respect to HPV frequencies [4,26].

It is noteworthy that Mexico is a country with a high incidence of CC [27]; however, in the present work we show a low percentage of mucosal HPV sequences, mainly HPV 16 (22%) in conjunctival carcinoma. Interestingly, limbus tumor was found related with the presence of HPV; this association could be explained because limbus tissue is quite similar in concept to that of transitional epithelium, as in cervical epithelium. The low frequency of HPV DNA in conjunctival cancer in our Mexican population failed to support HPV as a relevant etiological factor for this tumor type. Additional studies will be required prior to there being a definitive relationship between HPV and conjunctival cancer.

## Methods

### Tissues and DNA extraction

We analyzed 36 CSCC formalin-fixed paraffin-embedded specimens for HPV DNA detection. The samples were collected (during 2004-2008) from the Department of Pathology archives of the Hospital de la Luz, S.S., at Mexico City. All patients were subjected to surgery. All the samples consisted of the invasive conjunctiva squamous cell carcinoma. At present, the patients abandoned the clinical assistance, because they were free-disease. A tissue section of each specimen was hematoxilin and eosin stained and blindly analyzed by two independent pathologists to confirm the diagnosis. Information of clinical variables such as age, gender, sexually transmitted diseases, subtype clinical, histological differentiation and pterygium history were collected; in some cases the information were not available. Two 10 μm non-stained tissue sections were mounted on clean slides deparaffinized and rehydrated by standard methods. To avoid false negatives, defined tumor areas were manually microdissected under the light microscope (20× objective) with an sterilized needle, the scrapped tissue was then collected in a microtube containing 500 μl of digestion buffer (100 mM NaCl, 10 mM Tris-Cl pH 8, 25 mM EDTA pH 8, 0.5% SDS, and 0.1 mg/ml proteinase K), and incubated at 55°C for 48 hours. The DNA was extracted by means of Wizard Extraction kit (Promega, Madison WI, USA) according to manufacturers.

### HPV detection and typing

The presence of HPV DNA was determined by PCR analysis with consensus primers HPV16/E6 [28], which amplify, a fragment of E6 gene of 126 bp of HPV16. The PCR solution contained: 200 ng of tumor DNA, 1× buffer (50 mM KCl, 10 mM Tris- HCl, 0.1% Triton X-100), 2 mM MgCl_2_, 0.2 mM of each dNTP, 50 pmol of each primer and 2 units of Taq DNA polymerase (Promega) in 50 μl of final volume. The reaction tubes were placed in a thermal cycler (MJ Research Minicycler) with the following program: one cycle of denaturing at 94°C for 30 sec, and 40 cycles of denaturation at 94°C for 4 min, annealing at 55°C for 1.5 min, and extension at 72°C for 1.5 min, with a final extension at 72°C for 1.5 min. CaSKi DNA (HPV16+) was used as a positive control, C33 (HPV-), and lymphocyte DNA were included as negative controls. Before HPV detection, primers for human D-loop mitochondrial region genes were used as internal controls to monitoring DNA quality.

### Statistical analysis

All comparisons for significance were performed by means Χ^2^-test. All p values represent two-tailed test and were considered significant at 0.05. The statistical analysis was performed using the SPSS v15 statistical software.

## Competing interests

The authors declare that they have no competing interests.

## Authors' contributions

RP, AV and MS conceived and designed the study, analyzed data and drafted the manuscript, PE, VV, MM, FJ and DM helped with DNA extraction and PCR, CP, ET, LV and MT to provide biological samples and clinical data. All authors read and approved the final manuscript.
